# Phase II study of ramucirumab and docetaxel for previously treated non‐small cell lung cancer patients with malignant pleural effusion: Protocol of PLEURAM study

**DOI:** 10.1111/1759-7714.13279

**Published:** 2019-12-18

**Authors:** Shinnosuke Takemoto, Minoru Fukuda, Hiroyuki Yamaguchi, Takaya Ikeda, Kazumasa Akagi, Hiromi Tomono, Yasuhiro Umeyama, Yosuke Dotsu, Hirokazu Taniguchi, Hiroshi Gyotoku, Hiroaki Senju, Takeshi Kitazaki, Katsumi Nakatomi, Seiji Nagashima, Masaaki Fukuda, Akitoshi Kinoshita, Hiroshi Soda, Hiroshi Mukae

**Affiliations:** ^1^ Department of Respiratory Medicine Nagasaki University Graduate School of Biomedical Sciences Nagasaki Japan; ^2^ Clinical Oncology Center Nagasaki University Hospital Nagasaki Japan; ^3^ Department of Thoracic Oncology National Cancer Center Hospital East Kashiwa Japan; ^4^ Department of Respiratory Medicine Nagasaki Prefecture Shimabara Hospital Shimabara Japan; ^5^ Department of Respiratory Medicine Sasebo City General Hospital Sasebo Japan; ^6^ Department of Respiratory Medicine National Hospital Organization Nagasaki Medical Center Ohmura Japan; ^7^ Division of Respiratory Diseases, Department of Internal Medicine Japanese Red Cross Nagasaki Genbaku Hospital Nagasaki Japan; ^8^ Department of Respiratory Medicine National Hospital Organization Ureshino Medical Center Ureshino Japan

**Keywords:** docetaxel, NSCLC, pleural effusion, ramucirumab

## Abstract

**Introduction:**

Anti‐vascular endothelial growth factor therapy has been shown to be effective in non‐small cell lung cancer (NSCLC) patients with malignant pleural effusion (MPE); however, there are no data to suggest that ramucirumab has the same effects.

**Methods:**

We therefore decided to conduct a phase II study of ramucirumab plus docetaxel for NSCLC patients with MPE. The MPE control rate at eight weeks after the start of treatment will be the primary endpoint, and the objective response rate, progression‐free survival, one‐year survival rate, overall survival, and toxicity profile will be secondary endpoints.

**Discussion:**

A previous study indicated that administering chemotherapy in combination with bevacizumab was effective at controlling pleural effusion in patients with NSCLC with carcinomatous pleurisy. It is expected that ramucirumab will have a similar effect to the same group.

## Introduction

In Japan, 76 879 males and 35 739 females were afflicted with lung cancer in 2014, making it the third most common type of cancer in that year.^1^ In 2018, 55 100 males and 22 400 females died of lung cancer (the most common cause of cancer death).^1^ In recent years, the age‐adjusted mortality rate of lung cancer has gradually, but steadily, decreased in males while remaining the same in females.^2^

The prognosis of lung cancer patients with malignant pleural effusion (MPE) due to carcinomatous pleurisy is said to be poor. An investigation into the clinical effects of the accumulation of MPE in 490 lung cancer patients reported that 40% of patients had MPE and that the overall survival (OS) of patients with MPE was 5.5 months. Furthermore, 79 out of 94 patients (84%) with MPE underwent treatment for MPE (pleural effusion drainage or pleural effusion catheter placement) to relieve their symptoms, and it was reported that palliative MPE treatment was necessary if the volume of MPE was equivalent to >50% of the volume of the thoracic cavity.^3^ The Lung Cancer Diagnosis and Treatment Guidelines of the Japan Lung Cancer Society recommend pleurodesis for carcinomatous pleurisy that has been treated with thoracic cavity drainage. Meta‐analyses comparing various drugs showed that talc controlled MPE better than bleomycin, doxycycline, and tetracycline.^4^ Since the approval of the talc suspension method in 2013, talc, which has been shown to be able to control MPE, has been used universally in Japan. However, performing pleurodesis can cause patients' performance status (PS) to worsen, prolong sustained drainage, and delay the introduction of systemic drug therapy. Thus, effective and safe treatments for MPE are needed.

It has been reported that bevacizumab, an antivascular endothelial growth factor (VEGF) antibody, was effective in non‐small cell lung cancer (NSCLC) patients with MPE in two randomized studies in Japan.^5^, ^6^ However, there are no data to suggest that ramucirumab, an anti‐VEGF antibody that is used in the clinical setting, has the same effect. We decided to conduct a phase II, multicenter, single‐arm interventional study to evaluate the effectiveness and safety of ramucirumab as a treatment for MPE.

## Methods

### Objectives

The primary objective of this study is to evaluate the MPE control rate at eight weeks after the start of treatment with ramucirumab in combination with docetaxel (ramucirumab + docetaxel) in previously treated NSCLC patients with MPE.

The secondary objectives of the study are to evaluate the efficacy of ramucirumab + docetaxel, in terms of its effects on the objective response rate, progression‐free survival (PFS), one‐year survival rate, and OS, as well as its toxicity profile.

### Study design

The study protocol was reviewed and approved by Nagasaki University Clinical Research Review Committee (CRB7180001) (registration No. jRCTs071190013).

#### Clinical hypothesis and phase setting of the study

**Figure 1:**
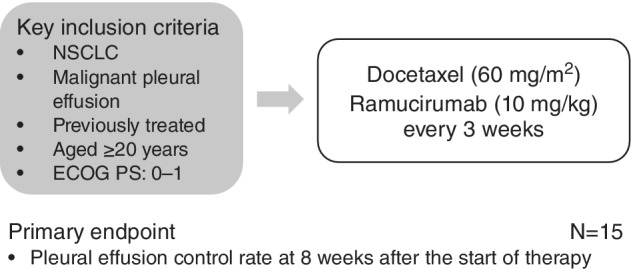
Study schema.

The clinical hypothesis underlying this study is that, “Combined treatment using docetaxel and ramucirumab is safe, even for NSCLC patients with MPE, and will show a certain ability to control pleural effusion”. Therefore, this will be a phase II study which examines the effects and safety of administering ramucirumab + docetaxel to NSCLC patients with MPE (Fig. 1). This study was sponsored by Eli Lilly Company.

### Inclusion and exclusion criteria

Inclusion criteria: (i) The patient has provided written consent after receiving a sufficient explanation about the study prior to enrollment; (ii) The patient is ≥20 years old on the day of enrollment; (iii) The patient has histologically‐ or cytologically‐confirmed NSCLC; (iv) The patient has clinical stage IV disease; (v) The patient exhibited disease progression during or after prior treatment with one (and only one) platinum‐based chemotherapy regimen with or without maintenance therapy for advanced/metastatic disease or in combination with an immune checkpoint inhibitor; (vi) The patient has clinical MPE and did not undergo pleurodesis after the discontinuation of the prior treatment; (vii) The patient does not have symptomatic superior vena cava syndrome; (viii) The patient is not suffering from invasion or narrowing of the major blood vessels due to cancer, according to documented radiological evidence; (ix) At least seven days have passed since the completion of radiotherapy to relieve the symptoms of metastatic lesions. At least 28 days must have passed if the radiotherapy field used to achieve symptom relief extended to the chest; (x) The patient has an Eastern Cooperative Oncology Group (ECOG) PS of 0 or 1 at the time of enrollment. and (xi) The patient has adequate organ function.

Patients will be excluded from the study if they meet any of the following exclusion criteria: (i) The patient requires treatment for MPE, such as urgent and continuous pleural effusion drainage due to displacement of the mediastinum caused by the accumulation of MPE (for reference: a blood oxygen saturation level [SpO_2_] of <90 Torr, unbearable respiratory difficulties, or ≥ 3 L of pleural effusion); (ii) The patient has undergone major surgery within the 28 days prior to enrollment or has undergone subcutaneous venous access device placement within the seven days prior to enrollment; (iii) The patient is receiving another concurrent anticancer treatment, including chemotherapy, immunotherapy, or targeted therapy; (iv) The patient has brain metastases that are symptomatic or require surgery, medication, or radiotherapy (except for stereotactic irradiation). Patients with treated brain metastases are eligible if they have clinically stable neurological functions and stopped taking steroids at least 14 days prior to enrollment if they underwent cranial irradiation (whole‐brain radiotherapy, focal radiotherapy, or stereotactic radiosurgery) or at least 28 days prior to enrollment if they underwent surgical resection; (v) The patient exhibits evidence of grade ≥ 1 central nervous system hemorrhaging on pretreatment magnetic resonance imaging (MRI) or an intravenous contrast‐enhanced computed tomography (CT) scan; (vi) The patient displays radiologically documented evidence of major blood vessel invasion or encasement by cancer; (vii) The patient exhibits radiological evidence of intratumor cavitation, regardless of the histology of the tumor; (viii) The patient has experienced an arterial thromboembolic event, including but not limited to a myocardial infarction, a transient ischemic attack, a cerebrovascular accident, or unstable angina, within the six months prior to the first dose of the protocol therapy being administered; (ix) The patient is receiving therapeutic anticoagulation with warfarin, low‐molecular‐weight heparin, or similar agents; (x) The patient experienced hemoptysis within the two months prior to enrollment; (xi) The patient has clinically relevant congestive heart failure (New York Heart Association class II–IV) or cardiac arrhythmia that is poorly controlled by medication; (xii) The patient has uncontrolled arterial hypertension of ≥150/≥90 mmHg, despite standard medical management; (xiii) The patient suffered a serious or non‐healing wound, ulcer, or bone fracture within the 28 days prior to enrollment; (xiv) The patient experienced a significant bleeding disorder, vasculitis, or grade 3/4 gastrointestinal (GI) bleeding within the three months prior to enrollment; (xv) The patient suffered GI perforation and/or GI fistula formation within the six months prior to enrollment; (xvi) The patient has a history of bowel obstruction, inflammatory enteropathy, extensive intestinal resection (hemicolectomy or extensive small intestine resection with chronic diarrhea), Crohn's disease, ulcerative colitis, or chronic diarrhea; (xvii) The patient has ≥grade 2 (according to the National Cancer Institute's Common Terminology Criteria for Adverse Events [NCI‐CTCAE] v4.0) peripheral neuropathy or the patient has a serious illness or medical condition(s); (xviii) The patient was curatively treated for previous or concurrent malignancies, except for basal or squamous cell skin cancer, in situ carcinoma of the cervix, or other solid tumors, and has not exhibited any evidence of recurrence for at least 3 years; (xix) The patient has preexisting interstitial lung disease, as evidenced by a chest CT scan and/or X‐ray at the baseline. (xx) The patient has serious preexisting medical conditions or serious concomitant systemic disorders that would compromise the safety of their patient or their ability to complete the study (at the discretion of the investigator); (xxi) The patient is pregnant (confirmed within seven days prior to enrollment) or breastfeeding; and (xxii) The patient has previously been treated with ramucirumab and/or docetaxel.

### Enrollment

Subjects who meet all of the criteria and have given their informed consent will be evaluated by the investigators for enrollment. The study doctor or his/her collaborator will carry out the enrollment by entering the data from the case enrollment forms and submitting it to the secretariat.

### Interventions

Thoracentesis: Prior to the start of treatment, pleural effusion will be drained as much as possible via thoracentesis. If the pleural effusion begins to reaccumulate, drainage via thoracentesis is permitted up until day 14 after the start of treatment.

Ramucirumab plus docetaxel combined treatment: Ramucirumab (10 mg/kg) plus docetaxel (60 mg/m^2^) will be administered every three weeks. The treatment will be continued until the criteria for treatment discontinuation are met. Ramucirumab will not be administered as a monotherapy. It is predicted that febrile neutropenia will occur often during this treatment, and pegylated granulocyte colony‐stimulating factor (PEG‐G‐CSF) preparations shall be used to treat such episodes.

### Outcomes

The tumor‐shrinking effects of the protocol treatment shall be judged using a procedure that follows the “New Response Evaluation Criteria In Solid Tumors (Revised RECIST Guidelines, version 1.1)“.^7^

The pleural effusion control rate at eight weeks after the start of treatment: With the total number of treated cases (excluding dropouts) being used as the denominator, the proportion of cases that do not require pleural effusion puncture or pleural effusion drainage within the first eight weeks after the start of treatment will be determined. The investigator must make a clinical judgment regarding whether pleural effusion puncture or pleural effusion drainage is required based on each patient's clinical findings, chest X‐rays, and CT scans.

The percentage of subjects that required treatments such as continuous pleural effusion drainage by day 21 after the start of treatment will also be calculated.

Overall survival (OS): OS is defined as the duration of the period from the date of enrollment to the date of death from various causes.

Progression‐free survival (PFS): PFS is defined as the duration of the period from the date of enrollment to the date of confirmed progression or death from various causes, whichever occurs first.

Response rate: The response rate is defined as the proportion of patients for whom the best overall effect is either a complete response or partial response.

Frequency of adverse events (adverse reactions): With the total number of treated cases being used as the denominator, the frequency of the worst grade (by group) of each adverse event (toxicity) throughout the course of treatment will be determined using the NCI‐CTCAE v4.0.

### Data monitoring and management

Periodic monitoring: This study will be monitored to ensure that it is being implemented safely and in accordance with the study protocol and that the data is collected accurately. A separate manual will outline the specific procedures used for the periodic monitoring.

Site visits and audits: In this study, there will be site visits and audits to ensure the reliability of the collected data and the integrity of the clinical research and protect the clinical research subjects. Site visits and audits will involve the auditor who will be appointed by the researching physician, visiting the study sites and checking the approval documents of the medical institution, the list of subinvestigators at the study site, and informed consent forms, and comparing and checking the case report form data against the information in the patients' medical charts (they will have direct access to the source material). The auditor will submit an Audit Report which will summarize the results of the audit to the researching physician/secretariat and investigators.

### Statistical considerations

The primary endpoint of this study is the MPE control rate at eight weeks.

Sample size: Based on an expected pleural effusion control rate of 50% and a threshold pleural effusion control rate of 20%, calculations performed using the one‐sided exact test with an α‐value of 0.05 and a β‐value of 0.20 showed that 13 subjects are needed. In anticipation of some patients dropping out, the required number of enrolled subjects was set at 15.

An interim analysis involving six patients will be conducted to analyze the safety of the protocol treatment.

Safety: The proportion of cases that require treatments such as continuous pleural effusion drainage by day 21 after the start of treatment will be calculated.

Pleural effusion control rate: The pleural effusion control rate at eight weeks after the start of treatment will be calculated.

PFS: The Kaplan‐Meier method will be used to estimate survival curves, median survival, yearly survival, etc.

Response rate: The response rate will be calculated.

Frequency of adverse events: The frequency of adverse events will be calculated.

This study may be discontinued if there are adverse events, enrollment problems, or for other reasons.

## Discussion

Two previous reports have indicated that administering cytotoxic anticancer agents, instead of performing pleurodesis, is effective against pleural effusion. In a phase II study in which 28 subjects were treated with carboplatin + pemetrexed + bevacizumab, the pleural effusion control rate was 93%,^5^ and in a phase II study in which 23 subjects were treated with carboplatin + paclitaxel + bevacizumab, the pleural effusion control rate was 87%.^6^ In the former study, anemia (CTCAE grade 3 or higher) was reported in 25% of subjects, while in the latter febrile neutropenia was seen in 26% of cases. The frequency of adverse events was higher in these studies than in the POINTBREAK study and ECOG4599 study. The latter studies found that the anti‐VEGF antibody bevacizumab, which is an angiogenesis inhibitor, reduced vascular permeability and pleural effusion. If pleural effusion can be controlled using systemic drug therapy without performing pleurodesis, it might be possible to avoid the worsening of PS associated with prolonged sustained drainage and delays in the introduction of systemic drug therapy.

In a phase III study that compared ramucirumab, a VEGF receptor 2 antibody that inhibits tumor angiogenesis, + docetaxel with docetaxel monotherapy, as a second‐line treatment (the REVEL study), the median OS in the ramucirumab + docetaxel group and docetaxel monotherapy group was 10.5 months and 9.1 months, respectively, which indicated that ramucirumab has synergistic effects (hazard ratio [HR]: 0.86, 95% confidence interval [CI]: 0.75–0.98).^8^ A randomized Japanese phase II study that compared ramucirumab + docetaxel with docetaxel monotherapy (the JVCG study) also showed that PFS tended to be longer in the ramucirumab + docetaxel group (5.2 months) than in the docetaxel monotherapy group (4.2 months) (HR: 0.83, 95% CI: 0.59–1.16).^9^ A response to treatment was seen in 28.9% of the ramucirumab + docetaxel group and 18.5% of the placebo group, and ramucirumab + docetaxel had a highly potent tumor‐shrinking effect. In terms of toxicity, the frequency of febrile neutropenia was higher in the ramucirumab + docetaxel group (34% vs. 19%). While hypoalbuminemia, thrombocytopenia, stomatitis, nosebleeds, and proteinuria exhibited high frequencies in the same group, most of these events were lower than grade 2 in severity. There are presently no data concerning the safety of administering ramucirumab + docetaxel to patients with NSCLC and carcinomatous pleurisy. However, a previous study indicated that administering chemotherapy in combination with bevacizumab was effective at controlling pleural effusion in patients with NSCLC with carcinomatous pleurisy. As it also inhibits angiogenesis, ramucirumab might have a similar effect.

## Disclosure

S.T. received an honorarium from MSD K.K. and Chugai Pharmaceutical Co., Ltd. MiF received an honorarium from Ono Pharmaceutical Co., Ltd., MSD K.K. Chugai Pharmaceutical Co., Ltd., Daichi Sankyo Co., Ltd, Kyowa Kirin Co., Ltd., and Roche Diagnostics K.K., and research funding from AstraZeneca K.K., Eli Lilly Japan, MSD K.K. HY received an honorarium from AstraZeneca K.K., Boehringer Ingelheim Japan, Taiho Pharmaceutical C., Eli Lilly Japan K.K., and Bristol‐Myers Squibb Company, and research funding from Novartis Pharma K.K., Eli Lilly Japan K.K., Boehringer Ingelheim Japan, Inc. HM received an honorarium from MSD K.K., Pfizer Japan Inc., Boehringer Ingelheim Japan, Astellas Pharma Inc., AstraZeneca K.K., Shionogi & Co., Ltd., Daiichi Sankyo Co., Ltd, Taisho Pharma Co., Ltd., Meiji Seika Pharma Co., Ltd. SRL, Inc., Asahi Kasei Pharma Corporation., Eli Lilly Japan, Ono Pharmaceutical Co., Ltd., Kyorin Pharmaceutical Co., Ltd., Sumitomo Dainippon Pharma Co., Ltd., Taiho Pharmaceutical Co., Mitsubishi Tanabe Pharma Corporation, Chugai Pharmaceutical Co., Teijin Home Healthcare Ltd, Toa Shinyaku Co., Ltd., Nihon Pharmaceutical Co., Ltd, and Janssen Pharmaceutical K.K., and research funding from Meiji Seika Pharma Co., Astellas Pharma Inc., Ono Pharmaceutical Co., Ltd., Daiichi Sankyo Company, Taisho Pharma Co., Taiho Pharmaceutical Co., Chugai Pharmaceutical Co., Fujifilm Toyama Chemical Co., Ltd., Eli Lilly Japan, and Boehringer Ingelheim Japan. The other authors declare that they have no conflicts of interest.
